# Training of Oral and Maxillofacial Surgery Residents in Virtual Surgical Planning: A Feasibility Study Comparing Open-Source Freeware and Commercially Available Software for Mandibular Reconstruction with Fibula Free Flap

**DOI:** 10.3390/cmtr18010010

**Published:** 2025-02-03

**Authors:** Bert Rombaut, Matthias Ureel, Benjamin Van der Smissen, Nicolas Dhooghe, Renaat Coopman

**Affiliations:** 1 Department of Oral and Craniomaxillofacial Surgery, Ghent University Hospital, Corneel Heymanslaan 10, 9000 Ghent, Belgium; bert.rombaut@skynet.be (B.R.); renaat.coopman@uzgent.be (R.C.); 2 Swiss MAM Research Group, Department of Biomedical Engineering, University of Basel, 4123 Allschwil, Switzerland; 3 Department of Health Technology, Vives University, Doorniksesteenweg 145, 8500 Kortrijk, Belgium; benjamin.vandersmissen@vives.be; 4 Department of Plastic and Reconstructive Surgery, Ghent University Hospital, 9000 Ghent, Belgium; nicolas.dhooghe@uzgent.be

**Keywords:** virtual surgical planning, mandibular reconstruction, education, medical, graduate (MESH), open-source freeware

## Abstract

*Study Design:* This is an experimental feasibility study. *Objective:* The objective was to analyze the potential of open-source freeware (OSF) to train residents in virtual surgical planning (VSP) and compare this workflow with commercially available software (CAS). *Methods:* A workflow for mandibular reconstruction with a fibular free flap (FFF) was developed in 3D-Slicer^®^ and Blender^®^ and compared to our clinical workflow in Materialise Mimics Innovation Suite version 25 (Materialise InPrint^®^, ProPlan CMF^®^ and 3-Matic^®^). Five CMF residents, inexperienced in VSP, were trained to use both the OSF and CAS workflows and then performed four planning sessions on OSF and CAS. The duration (minutes) and the amount of mouse clicks (MCs) of every step in the workflow were recorded. Afterwards, the experience with VSP was investigated with the System Usability Scale (SUS) and a self-developed questionnaire. *Results:* The total VSP time with CAS took 91 ± 15 min and needed 2325 ± 86 MCs compared to 111 ± 26 min and 1876 ± 632 MCs for OSF, respectively. The questionnaire had an 80% response rate. The SUS for CAS was 67.5 compared to 50 for OSF. The participants believe it is extremely valuable to learn VSP during their training and to be able to perform VSP as a surgeon. *Conclusion:* We believe OSF can be a cost-effective alternative compared to CAS for the training of surgical residents to gain insight in complex surgeries and to better understand CAD limitations and possibilities.

## 1. Introduction

In recent decades, several important technological innovations have been cemented in the daily clinical practice of oral and maxillofacial surgery (OMFS). In particular, virtual surgical planning (VSP) and Additive Manufacturing (AM) have become indispensable for complex surgical procedures. It has been shown that preoperative VSP can improve surgical accuracy, reduce operating time, and reduce surgical costs [1,2,3]. By using computer-aided design (CAD) software, patient-specific anatomical models and surgical cutting guides (SCGs) can be created. These AM tools make complex surgeries more predictable and more accurate, and result in reduced operating time [4,5].

For mandibular reconstructions with fibular free flap, the affected mandibular bone is removed and replaced with an osseous free flap for functionality, esthetics, and facial contouring [6]. With VSP, SCGs are designed based on the bony surfaces of the patient’s relevant anatomy and guide the surgeon’s saw or drill, immediately obtaining the correct angles and sizes of the osteotomy and resection parts [7,8].

The drawbacks of VSP are high costs, the planning time and preparation time required, and the need for additional training [9]. For the surgeon, there is a significant learning curve and time investment required to acquire the necessary skills, collect the data, perform the planning, and create the 3D models and cutting guides. It thus makes sense to outsource VSP and AM within the industry. However, this entails privacy issues, more processing costs, prolonged delivery time before surgery, and time-consuming meetings with partner companies for the development of VSP [4,5]. Also, innovative ideas and solutions to problems encountered intraoperatively are less likely to be implemented or solved by industry partners. Another important disadvantage is the price of CE- or FDA-approved commercially available software (CAS) packages. In the European Union (EU), medical devices, including software used for surgical planning, must comply with the requirements set forth in the Medical Devices Regulation (MDR) [10]. There are OSF packages available that can be used free of charge and that have been successfully used in patients without CE or FDA approval [11,12]. However, as correctly stated by Tel A. et al., these software packages are essentially not approved for medical use [13].

A logical answer to these problems is the implementation of in-house AM labs at the Point of Care (POC), as this could reduce costs and allow a more active role of the surgeon in the planning process [5].

The principles of surgical education have not changed, but methods for surgical teaching have evolved with time [14]. There is instant access to information, enabling residents to learn anywhere at any time. The incredible increase in the availability of new technologies, the reduction in working hours, and the high expectations of surgical trainees are transforming residents’ training [15]. The implementation of surgical and robotic simulators and virtual and/or augmented educative tools is well investigated in the literature, resulting in more confidence and better surgical preparation of the residents [16,17]. However, the literature about the use of virtual surgical planning by surgeons or young residents as a training and learning tool is scarce.

Due to the similarity of the principles of VSP used by OSF and CAS workflows, we believe that the use of OSF could be a valuable cost-effective method to train residents and surgeons in the principles of CAD. In this study, the feasibility of training inexperienced CMF residents to virtually plan a mandibular reconstruction with an FFF with open-source software compared to commercially available software (gold standard) is investigated.

## 2. Materials and Methods

Three exemplary patient cases with T4 oral squamous cell carcinoma (OSCC) with mandibular invasion were selected for this study. All cases received a segmental resection of the mandible and immediate reconstruction with a vascularized FFF. All cases were preoperatively planned with Materialise Mimics Innovation Suite^®^ v25 software (Leuven, Belgium) by the surgeons R.C. and M.U. According to the Declaration of Helsinki, written informed consent of the patients was obtained.

### 2.1. Software Packages

A commercial software package, the Materialise Mimics Innovation Suite^®^ version 25, was used. This software package is CE-marked and FDA-approved for medical use. Therefore, the Materialise^®^ software is considered the gold standard in this study.

Materialise InPrint^®^ was used for segmentation of the DICOM images, Materialise ProPlan CMF^®^ for the planning of the mandibular and fibular osteotomies and mandibular reconstruction, and Materialise 3-Matic^®^ for the design of the surgical cutting guides.

An OSF 3D-Slicer^®^ (Earth, TX, USA) was used for the segmentation of the DICOM images and Blender^®^ (Amsterdam, The Netherlands) for the remaining steps of the workflow. 3D-Slicer^®^ is a free, open-source multi-platform software package widely used for medical, biomedical, and related imaging research [18]. Blender^®^ is a free and open-source multi-platform community-driven 3D creation suite [19].

### 2.2. VSP Workflow Principles in Mandibular Reconstruction

**Figure 1:**
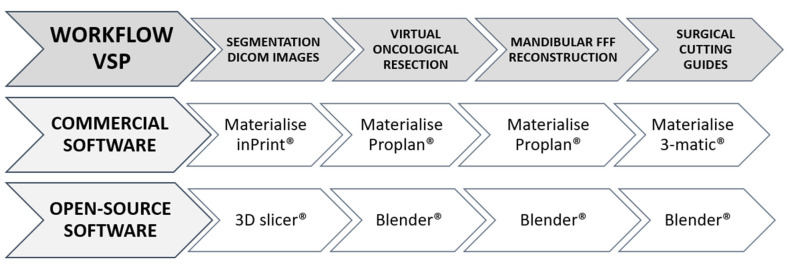
Workflow VSP. **Upper line**: from left (L) to right (R): 4 steps in the VSP workflow: segmentation DICOM images, virtual oncological resection, mandibular FFF reconstruction, and SCG design. Middle line: CAS software workflow (Mimics InPrint^®^, ProPlan CMF^®^, 3-matic^®^). **Bottom line**: OSF software workflow (3D Slicer^®^, Blender^®^). (*VSP **=** virtual surgical planning*; *CAS* = *commercially available software*; *OSF* = *open-source freeware*).

The VSP was divided into four planning steps: (i) DICOM image segmentation; (ii) virtual oncological resection; (iii) mandibular FFF reconstruction; and (iv) design of surgical cutting guides (Figure 1).

#### 2.2.1. DICOM Image Segmentation

DICOM images of CT scans consist of Hounsfield units (HU), which do not contain anatomical information. The segmentation process highlights anatomical regions as regions of interest (ROIs) on the DICOM images. In the case of mandibular reconstruction, a threshold of 500–3000 HU was used. After removal of artifacts created after thresholding, the segmented or colored voxels were divided into anatomical structures. These ROIs were then optimized and transformed into surface tessellation language (STL) files. In the case of a mandibular reconstruction with a FFF, the cranium, mandible and fibula were segmented. After segmentation, virtual anatomical models of the patients were generated and used for VSP.

#### 2.2.2. Virtual Oncological Resection and Reconstruction

There are two major phases in the mandibular reconstruction planning. First, the osteotomy planes of the mandibular resection are determined by the oncological margins (virtual oncological resection). Second, the placement and orientation of the FFF in the remaining parts of the mandible (mandibular reconstruction) are carried out.

Following the segmentation process, the virtual oncological resection was designed at the defined surgical resection margins on the anatomical parts of the skull. The osteotomies were constructed by planes, determined by three selected points on the mandible. After adaptation of the position and orientation, perpendicular to the mandible, the osteotomies were applied separating the mandible into healthy and oncological parts.

**Figure 2:**
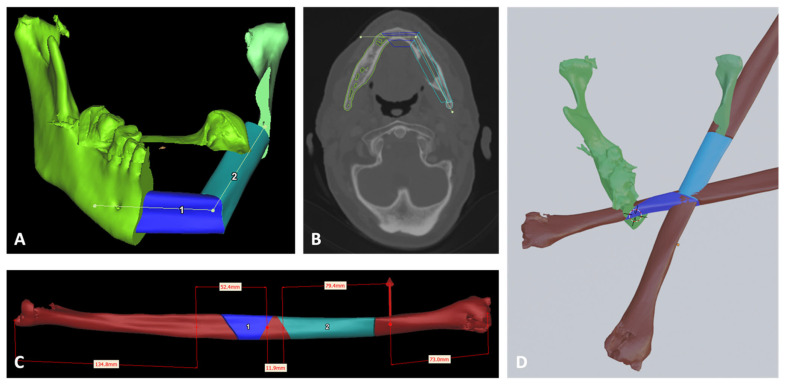
Screenshots during VSP. (**A**): Segmented mandible after virtual oncological resection with positioning of the fibula free flap segments using Proplan CMF. (**B**): The segmentation and the position of the fibula parts can be checked on the 2D DICOM images in Proplan CMF. (**C**): Visualization of the fibula and the fibula segments used for mandibular reconstruction in Proplan CMF. The distances of each part and the distance from the malleolus are annotated. (**D**): Positioning of the fibula parts using Blender.

Next, the mandibular reconstruction with FFF was planned (Figure 2A). In Proplan CMF^®^, a reconstruction line is drawn on the healthy parts of the osteotomized mandible and the software automatically calculates the dimensions and position of the fibula parts, considering the distance to the malleolus process (7 cm) and in between the fibula parts (Figure 2C). The fibula parts can be repositioned, rotated and/or the vascularization inverted. Visual verification on the original DICOMs is possible as shown in Figure 2B. In Blender^®^, the entire fibula must be positioned in the mandible (Figure 2D). Armatures are structures to which other objects can be attached and are used to rotate the fibula in the mandible. At the start, line segments are drawn to estimate the armature size. When a correct position of the fibula is found, the cutting planes are added to divide the fibula into segments. Afterwards, the segments are rotated back in their original fibular position for the SCG design.

#### 2.2.3. Design of Surgical Cutting Guides

The design of mandibular and fibular cutting guides follows similar processes. The surgeons in this study preferred the use of cutting boxes, which were designed by performing a wrap procedure of 2.0 mm around the defined osteotomy plane. A base plate (thickness of 2.0 mm) was designed with respect to anatomical landmarks (mandibular angle, tooth position and/or position of the malleolus). Both the cutting box and base plate were merged, and the osteotomy plane and anatomical structures subtracted with a clearance of 0.3 mm.

### 2.3. Study Design

An inexperienced engineering student (B.R.) was trained to use the Materialise Mimics Innovations Suite^®^ version 25, 3D-Slicer^®^ and Blender^®^ by surgeon R.C. using available guidelines, hands-on lessons, online videos, software manuals, and visits to the operating theater to better understand the advantages and limitations of the surgery. B.R. produced practical written guidelines and educational videos for the use of the CAS and OSF workflow (See Supplementary Materials, https://www.youtube.com/@rombaut4678 (accessed on 11 January 2025)).

Five CMF residents, all inexperienced in VSP, volunteered to participate and received educational training from B.R. with the gold standard (Materialise Mimics InPrint^®^, ProPlan CMF^®^, 3-Matic^®^) and the OSF software (3D-slicer^®^, Blender^®^), using the abovementioned instructional videos, software manuals, hands-on lessons, and the written practical guidelines.

After the educational training, all participants performed an initial training session, followed by 3 performing sessions with CAS and OSF. The performing sessions took place in groups, i.e., 5 CMF residents and 1 instructor (B.R.), and were organized twice a week (one session for OSF and one session for CAS), every week for 3 weeks. Each week, another oncological case was used with a total of 3 oncological cases. All the abovementioned learning materials were available to use during the training and performing sessions.

During the training and performing sessions, the four planning steps were monitored and compared in terms of duration (number of minutes required per step) for the training session and duration combined with efficiency (number of mouse clicks (MCs) for performing sessions. The duration was measured with a standard stopwatch of an iPhone SE 2020 series (Apple^®^, Cupertino, CA, USA), and the MCs were tracked and recorded with a digital counter (Click Counter^®^, SourceForge, San Diego, CA, USA). The average of the duration and number of mouse clicks was calculated per step per session to simplify analyses. During the training session, all VSPs were performed step-by-step following B.R., meaning that all mouse clicks were the same between the CMF residents.

### 2.4. Questionnaire

After the performing sessions, each participant received a questionnaire via email evaluating their experiences and usability of the CAS and OSF workflow. The standardized System Usability Scale (SUS) is a 10-item questionnaire with 5 response options in the form of a Likert scale and was used to evaluate the usability of the workflow [20]. Each answer corresponds to a score of 1–5, and after scoring, a result between 0 and 100 is obtained. A score above 68 would be considered above average [21]. Additionally, some self-developed questions (Q11–14) were asked to learn about the experience of the residents with VSP. Questions were in the form of a 5-point Likert scale and visualized in bar charts. The questionnaires were collected by an impartial contact person and anonymized for the investigators.

### 2.5. Statistical Analysis

Data were collected and analyzed in Microsoft^®^ Excel (Redmond, WA, USA). The questionnaire is added to this manuscript as a Supplementary Materials. Statistical analysis of the available data using a non-parametric Wilcoxon signed-rank test was performed. A *p*-value lower than 0.05 was considered statistically significant. Statistically non-significant results were marked with NS.

## 3. Results

### 3.1. Descriptive Analysis of Oncological Cases

Table 1 includes population parameters of the three exemplary oncological cases used in this study.

### 3.2. Study Results

All five participants were able to complete VSP in all cases. Due to differences in computational power of the hardware, it was not possible to make an objective evaluation of the first planning step (DICOM image segmentation) resulting in the exclusion of these results. During the training session, the mouse clicks were not recorded.

**Figure 3:**
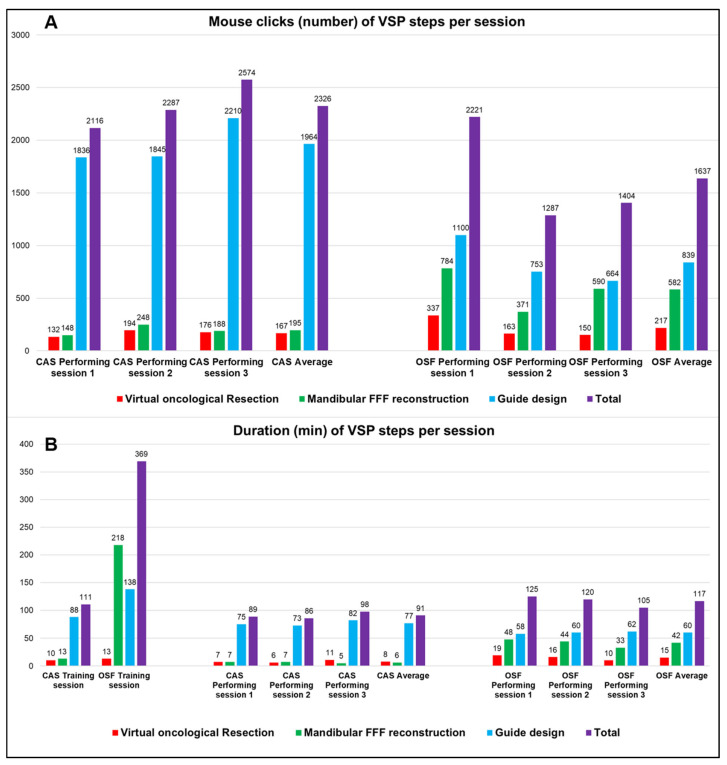
(**A**): Overview of the efficiency per session for each of the planning steps expressed in number of mouse clicks. (**B**): Overview of the duration per session for each of the planning steps expressed in minutes. (*VSP* = *virtual surgical planning*; *CAS* = *commercially available software*; *OSF* = *open-source freeware*; *FFF* = *fibula free flap*).

The original data are reported in Table 2 and visualized in Figure 3. The duration of the virtual oncological resection step of the initial training session is comparable with CAS and OSF, 10 and 13 min, respectively, but for the mandibular reconstruction and guide design, OSF needed 218 and 138 min compared to 13 and 88 min for CAS.

In the performing sessions, the virtual oncological resection and mandibular reconstruction steps were faster with CAS, but the design of the surgical guides seemed to be comparable but significantly lower, with an average of 77 min (SD 4.7 min) and 60 min (SD 2.0 min), respectively (*p* < 0.05). In total, planning time of CAS was on average 91 min (SD 6.2 min), compared to 117 min (SD 10.4 min) with OSF (*p* < 0.05). In terms of efficiency, more mouse clicks are needed for the surgical guide design when using CAS, 1964 MCs (213.4 MCs) on average, compared to 839 MCs (230.4 MCs) for OSF (*p* < 0.001). Total number of mouse clicks were lower with OSF than CAS, 1637 MCs (SD 508.8 MCs) compared to 2326 MCs (231.4 MCs) (*p* < 0.001).

Our results show that OSF has a steep learning curve as the participants needed 369 min compared to 111 min for CAS to finish the training session. However, during the performing sessions, we noticed a strong decline in duration of the OSF workflow, leading to an average of 117 min (SD = 10.4) per VSP, compared to 91 min (SD = 6.2) for CAS (*p* = NS). In terms of efficiency, the results show that CAS needed a higher number of MCs to complete a VSP compared to OSF, 2326 (SD = 231.4) and 1637 (SD = 508.8).

### 3.3. Questionnaire

**Figure 4:**
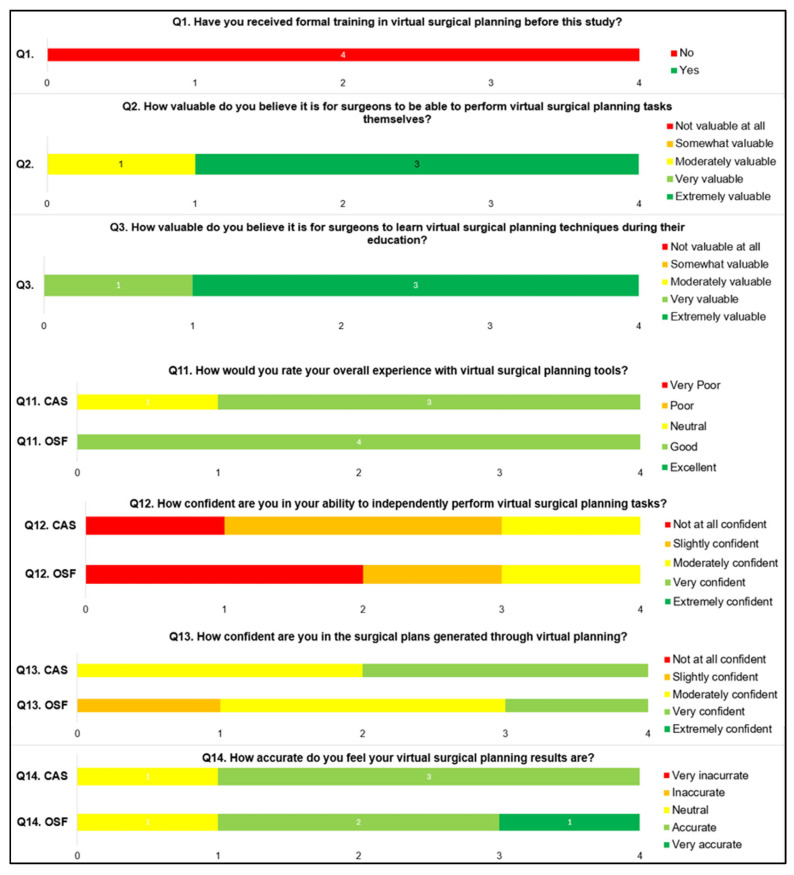
Overview of all answers to the self-developed questionnaire. A total of 4 out of 5 participants completed the questionnaire. (*OSF* = *open-source freeware*; *CAS* = *commercially available software*).

Four out of five participants completed the questionnaire (Q2–Q10) resulting in an SUS score of 67.5 (SD 12.4) for the CAS workflow and 50 (SD 11.0) for the OSF workflow (*p* < 0.05), showing preference for the CAS workflow. None of the participants had received previous training in VSP, and all participants reported a neutral to good experience. Most participants believed that learning VSP during their education and being able to perform VSP as a surgeon is moderately to extremely valuable. The residents were confident in the created surgical plans through virtual planning and felt that they were accurate, but did not feel confident to perform the VSP independently. All answers to the self-developed questionnaire (Q1, Q11–Q14) are visualized in Figure 4.

## 4. Discussion

This study aims to investigate the feasibility of OSF as an inexpensive VSP training tool for CMF surgery residents, using mandibular reconstruction with FFF as an example. The objectives of this study were threefold: firstly, to demonstrate that OSF could serve as an alternative to CAS, thereby democratizing virtual surgical planning (VSP) globally; secondly, to show that the OSF method is teachable to residents; thirdly, to demonstrate that while OSF has a learning curve compared to CAS, this curve can be significantly reduced after a few attempts.

In the literature, there are no studies comparing open-source freeware and commercially available software for the planning of mandibular reconstruction with osseomyocutaneous free flaps. There are several studies describing VSP of mandibular reconstruction with different software packages and different designs [12,22,23,24,25]. This study aims to investigate the feasibility of OSF as a free educational tool for CMF residents compared to CAS, in this case Materialise Innovation Suite version 25, the most widely used software package in CMF surgery worldwide [26].

During the study, CMF residents needed more time to get used to the control mechanisms of Blender^®^ and had to learn short keys, leading to a steeper learning curve. Also, the use of hinges and defining the osteotomy planes in the fibula is quite time-consuming in the beginning. After the initial training session, a strong reduction in the time spent on mandibular reconstruction and designing time of SCGs was observed. Our time estimates are similar to the reported planning times of 156 min on average for the first 5 cases and 79 min on average for the following 14 cases by Abo Sharkh et al. [11]. The efficiency of the workflows expressed in the number of mouse clicks is higher for resection and reconstruction in the CAS workflow, explained by the specifically developed workflow in CMF Proplan^®^. However, we noticed that the design of the cutting guides requires less mouse clicks in Blender^®^ compared to 3-Matic^®^.

Blender^®^ proved to be a viable alternative for the design of SCGs. But, in general, VSP for mandibular reconstruction with FFF leans in favor of CAS. Firstly, in OSF, there is a lack of DICOM image verification. Secondly, working with the armatures for the fibula is suboptimal compared to the automatic positioning of CAS. Thirdly, the angles and proportions of all parts are visualized in OSF but making mistakes leads to starting over from step one. The biggest advantage of CAS is the simplicity and user-friendliness. The use of clear patterns and functions makes the software very intuitive. This explains the higher score on the SUS, 67.5 compared to 50 for OSF.

Instructions on how to perform mandibular reconstructions with CAS and OSF are readily available on the internet. Some articles provide step-by-step videos on how to perform VSP on mandibular reconstruction [27]. These software packages are often updated providing new and more efficient tools (e.g., ‘snapping’ tool, ’shrink-wrap’ tool) allowing for immediate object fitting to other objects simplifying SCG design for planning. Especially for Blender^®^, the source code is publicly available, meaning that anybody can develop additions and improve the existing software. In the Supplementary Sections, an educational video explaining the basic controls of Blender^®^, giving the user a step-by-step protocol on how to create SCGs, was added.

This study has several limitations. The surgeon R.C. is used to working clinically with the CAS and to a lesser extent with OSF. As the training of B.R. was partly performed by R.C., this could have created a bias in the results. This study is based on a small study population of five CMF residents who voluntarily participated. As these residents are affiliated to our clinic, this article is prone to selection and reporting bias. Only four sessions, one training session and three performing sessions, were performed indicating that more sessions could result in faster and more efficient results. Only one OSF workflow was compared to the gold standard. There are other CAS and OSF workflows that could be useful as educational tools. The relevance of this article could be discussed as the technology is rapidly evolving. Artificial intelligence could have an enormous impact on VSP as segmentations and SCG could be automated. It is expected that, before 2030, there will be a steep increase in mixed and augmented reality applications, and surgical planning itself will be performed by virtual interaction with the physical space, manipulating objects and repositioning bones [26]. For this manuscript, the Materialise Innovation Suite version 25 was used. There is, however, a new release with a more efficient surgical guide design. The results of this study are thus dependent on the type and the version of the software.

The participants consisted of one female and four males. In general, they reported a good experience with the virtual surgical planning tools without having received any previous VSP training. They were, in general, confident about the accuracy of the planning results and seemed somewhat more confident in the plans generated by CAS compared to OSF. Interestingly, they did not feel confident in their ability to perform VSP independently. In this feasibility study, it became clear that the CMF residents find it extremely valuable for surgeons to have the opportunity to learn how to use VSP techniques during their education and to be able to perform VSP themselves. As all VSPs for mandibular reconstruction cases are performed by responsible surgeons, these results can be biased.

The authors acknowledge that the statistics and number of cases are limited. In Figure 4, we observe that, compared to the initial training session, the learning curve after approximately three VSP cases remained consistent for both CAS and OSF, with OSF taking slightly more time. The authors wish to add that the time investment required from our residents was high and their participation in this research project was completely voluntarily. We also noticed a partial decline in the residents’ enthusiasm to perform more sessions in the study. On the other hand, despite the limited number of cases, the performance and speed of VSP remained approximately the same after three cases, both for CAS and OSF. The question then arises whether increasing the number of cases in this study would provide added value in demonstrating feasibility.

Additionally, we would like to highlight the following:

1. This is one of the first studies to effectively compare OSF and CAS based on metrics such as time and mouse clicks, evaluated across multiple relevant stages of VSP (segmentation, virtual resection, reconstruction, and the creation of cutting guides).
2. This study features a fully developed YouTube channel, enabling the reproduction of every step. To our knowledge, such an approach has not been explored in other publications.
3. This study provides opportunities for young individuals to familiarize themselves with the technology without relying on clinical internships, which allows them to engage with the technology from an early stage.
4. This study offers the potential to adopt and implement this technology in regions with limited budgets.

Despite the limitations in sample size, hospital size, and resident availability, we believe that these objectives have not yet been published and that the approach is innovative. The feasibility of this method, although limited, has been demonstrated.

We believe that OSF can be a cost-effective training and educational tool for residents and beginning surgeons. Participation of the residents in planning real actual surgical cases and letting them participate during surgery are efficient ways to engage, challenge and empower residents. The possibility of in-house planning of complex surgeries engages the students and surgeons to prepare the case in detail, leading to a better comprehension and potentially a higher demand for the result [12]. Just as engineers are trained in surgical protocols, residents and surgeons should be trained in the limitations and, more importantly, the huge potential of VSP and AM. By introducing VSP training into the surgical curriculum, the residents could gain important insights in complex surgeries, learn how to interact with clinical engineers, understand the limitations of VSP and stay up to date with new technological advances.

## 5. Conclusions

This study examined the feasibility of performing a mandibular reconstruction with a fibular free flap using OSF (3D-Slicer^®^ and Blender^®^) compared to a gold-standard commercially available software package (Materialise^®^) as an educational tool for CMF residents. Our results indicate that virtual surgical planning with OSF is comparable in terms of duration, but the CAS package is more efficient and has certain workflow advantages. VSP is perceived as an extremely valuable skill as a surgeon by the participants. We believe OSF can be a cost-effective alternative compared to CAS for the training of surgical residents to gain insight in complex surgeries and to better understand CAD limitations and possibilities. Additional multi-center research projects should determine whether the implementation of VSP training modules in the existing surgical training programs would be beneficial.

## Figures and Tables

**Table 1:** Descriptive overview of the oncological cases. (M = male; F = female; OSCC = oral squamous cell carcinoma; L = left; R = right).

Sex	Age	Pathology	Resection	Reconstruction
M	72	OSCC	Corpus + L ramus	Fibula R
M	63	OSCC	Corpus + R ramus	Fibula L
F	75	OSCC	Corpus + R ramus and condyle	Fibula L

**Table 2:** Original data of the duration in minutes and number of mouse clicks of training and performing sessions with calculated averages and standard deviation between the brackets. (*min* = *minutes*; *CAS* = *commercially available software*; *OSF* = *open-source freeware*; *FFF* = *free fibular flap*, *NA* = *not applicable*; *NS* = *not significant*).

	Duration (Min)			Mouse Clicks (Number)		
	CAS	OSF	*p*-Value	CAS	OSF	*p*-Value
Training Session						
Virtual Resection	10	13	NS	NA	NA	NA
Virtual Resection	13	218	<0.001	NA	NA	NA
Fff Reconstruction	88	138	<0.001	NA	NA	NA
Cutting Guide Design	111	369	<0.001	NA	NA	NA
Performing Session 1						
Virtual Resection	7	19	<0.001	132	337	<0.001
Fff Reconstruction	7	48	<0.001	148	784	<0.001
Cutting Guide Design	75	58	<0.05	1836	1100	<0.001
Total	89	125	<0.001	2116	2221	NS
Performing Session 2						
Virtual Resection	6	16	<0.001	194	163	NS
Fff Reconstruction	7	44	<0.001	248	371	<0.05
Cutting Guide Design	73	60	NS	1845	753	<0.001
Total	86	120	<0.05	2287	1287	<0.001
Performing Session 3						
Virtual Resection	11	10	NS	176	150	NS
Fff Reconstruction	5	33	<0.001	188	590	<0.001
Cutting Guide Design	82	62	<0.05	2210	664	<0.001
Total	98	105	NS	2574	1404	<0.001
Average						
Virtual Resection	8.0 (2.6)	15.0 (4.6)	<0.001	167.3 (31.9)	216.7 (104.4)	<0.001
Fff Reconstruction	6.3 (1.2)	41.7 (7.8)	<0.001	194.7 (50.3)	581.7 (206.6)	<0.001
Cutting Guide Design	76.7 (4.7)	60.0 (2.0)	<0.05	1963.7 (213.4)	839.0 (230.4)	<0.001
Total	91.0 (6.2)	116.7 (10.4)	<0.05	2325.7 (231.4)	1637.3 (508.8)	<0.001

## Data Availability

The original contributions presented in this study are included in the article/Supplementary Materials. Further inquiries can be directed to the corresponding author.

## Supplementary Materials

The following supporting information can be downloaded at: educational videos https://www.youtube.com/@rombaut4678. The questionnaire can be downloaded at: https://www.mdpi.com/article/10.3390/cmtr18010010/s1, uploaded as Supplementary Materials.
