# Supplementary material for: Training of Oral and Maxillofacial Surgery Residents in Virtual Surgical Planning: A Feasibility Study Comparing Open-Source Freeware and Commercially Available Software for Mandibular Reconstruction with Fibula Free Flap
Source: Craniomaxillofac Trauma Reconstr. 2025 Feb 3;18(1):10. doi: 10.3390/cmtr18010010 (PMC11995818; doi:10.3390/cmtr18010010)
Supplement: Supplementary file 1 [file cmtr-18-00010-s001.zip › cmtr-3423872-supplementary.pdf]

Questionnaire:
Comparing two VSP workflows for mandible reconstruction with fibula free flap.

GENERAL QUESTIONS

1. Have you received formal training in virtual surgical planning before this study?

- [ ] Yes
- [ ] No

2. How valuable do you believe it is for surgeons to be able to perform virtual surgical planning tasks themselves?

- [ ] Not valuable at all
- [ ] Somewhat valuable
- [ ] Moderately valuable
- [ ] Very valuable
- [ ] Extremely valuable

3. How valuable do you believe it is for surgeons to learn virtual surgical planning techniques during their education?

- [ ] Not valuable at all
- [ ] Somewhat valuable
- [ ] Moderately valuable
- [ ] Very valuable
- [ ] Extremely valuable

PART 1: WORKFLOW CAS

1. I think that I would like to use this system frequently.

   - ☐ Strongly Disagree
   - ☐ Disagree
   - ☐ Neutral
   - ☐ Agree
   - ☐ Strongly Agree

2. I found the system unnecessarily complex.

   - ☐ Strongly Disagree
   - ☐ Disagree
   - ☐ Neutral
   - ☐ Agree
   - ☐ Strongly Agree

3. I thought the system was easy to use.

   - ☐ Strongly Disagree
   - ☐ Disagree
   - ☐ Neutral
   - ☐ Agree
   - ☐ Strongly Agree

4. I think that I would need the support of a technical person to be able to use this system.

   - ☐ Strongly Disagree
   - ☐ Disagree
   - ☐ Neutral
   - ☐ Agree
   - ☐ Strongly Agree

5. I found the various functions in this system were well integrated.

   - ☐ Strongly Disagree
   - ☐ Disagree
   - ☐ Neutral
   - ☐ Agree
   - ☐ Strongly Agree

6. I thought there was too much inconsistency in this system.

- ☐ Strongly Disagree
- ☐ Disagree
- ☐ Neutral
- ☐ Agree
- ☐ Strongly Agree

7. I would imagine that most people would learn to use this system very quickly.

- ☐ Strongly Disagree
- ☐ Disagree
- ☐ Neutral
- ☐ Agree
- ☐ Strongly Agree

8. I found the system very cumbersome to use.

- ☐ Strongly Disagree
- ☐ Disagree
- ☐ Neutral
- ☐ Agree
- ☐ Strongly Agree

9. I felt very confident using the system.

- ☐ Strongly Disagree
- ☐ Disagree
- ☐ Neutral
- ☐ Agree
- ☐ Strongly Agree

10. I needed to learn a lot of things before I could get going with this system.

- ☐ Strongly Disagree
- ☐ Disagree
- ☐ Neutral
- ☐ Agree
- ☐ Strongly Agree

11. How would you rate your overall experience with virtual surgical planning tools?

- ☐ Very Poor
- ☐ Poor
- ☐ Neutral
- ☐ Good
- ☐ Excellent

12. How confident are you in your ability to independently perform virtual surgical planning tasks?

- ☐ Not at all confident
- ☐ Slightly confident
- ☐ Moderately confident
- ☐ Very confident
- ☐ Extremely confident

13. How accurate do you feel your virtual surgical planning results are?

- ☐ Very inaccurate
- ☐ Inaccurate
- ☐ Neutral
- ☐ Accurate
- ☐ Very accurate

14. How confident are you in the surgical plans generated through virtual planning?

- ☐ Not at all confident
- ☐ Slightly confident
- ☐ Moderately confident
- ☐ Very confident
- ☐ Extremely confident

PART 2: WORKFLOW OSF

1. I think that I would like to use this system frequently.

   - ☐ Strongly Disagree
   - ☐ Disagree
   - ☐ Neutral
   - ☐ Agree
   - ☐ Strongly Agree

2. I found the system unnecessarily complex.

   - ☐ Strongly Disagree
   - ☐ Disagree
   - ☐ Neutral
   - ☐ Agree
   - ☐ Strongly Agree

3. I thought the system was easy to use.

   - ☐ Strongly Disagree
   - ☐ Disagree
   - ☐ Neutral
   - ☐ Agree
   - ☐ Strongly Agree

4. I think that I would need the support of a technical person to be able to use this system.

   - ☐ Strongly Disagree
   - ☐ Disagree
   - ☐ Neutral
   - ☐ Agree
   - ☐ Strongly Agree

5. I found the various functions in this system were well integrated.

   - ☐ Strongly Disagree
   - ☐ Disagree
   - ☐ Neutral
   - ☐ Agree
   - ☐ Strongly Agree

6. I thought there was too much inconsistency in this system.

- [ ] Strongly Disagree
- [ ] Disagree
- [ ] Neutral
- [ ] Agree
- [ ] Strongly Agree

7. I would imagine that most people would learn to use this system very quickly.

- [ ] Strongly Disagree
- [ ] Disagree
- [ ] Neutral
- [ ] Agree
- [ ] Strongly Agree

8. I found the system very cumbersome to use.

- [ ] Strongly Disagree
- [ ] Disagree
- [ ] Neutral
- [ ] Agree
- [ ] Strongly Agree

9. I felt very confident using the system.

- [ ] Strongly Disagree
- [ ] Disagree
- [ ] Neutral
- [ ] Agree
- [ ] Strongly Agree

10. I needed to learn a lot of things before I could get going with this system.

- [ ] Strongly Disagree
- [ ] Disagree
- [ ] Neutral
- [ ] Agree
- [ ] Strongly Agree

11. How would you rate your overall experience with virtual surgical planning tools?

- [ ] Very Poor
- [ ] Poor
- [ ] Neutral
- [ ] Good
- [ ] Excellent

12. How confident are you in your ability to independently perform virtual surgical planning tasks?

- ☐ Not at all confident
- ☐ Slightly confident
- ☐ Moderately confident
- ☐ Very confident
- ☐ Extremely confident

13. How accurate do you feel your virtual surgical planning results are?

- ☐ Very inaccurate
- ☐ Inaccurate
- ☐ Neutral
- ☐ Accurate
- ☐ Very accurate

14. How confident are you in the surgical plans generated through virtual planning?

- ☐ Not at all confident
- ☐ Slightly confident
- ☐ Moderately confident
- ☐ Very confident
- ☐ Extremely confident
